# Social Support as a Determinant of Dietary Quality in Community‐Dwelling Older Adults in China

**DOI:** 10.1002/fsn3.71476

**Published:** 2026-01-22

**Authors:** Xiaoyan Zhang, Yuanyuan Yan, Feika Li, Jialin Liu, Fang Wu, Dongsheng Bian

**Affiliations:** ^1^ Department of Geriatrics, Ruijin Hospital Shanghai Jiao Tong University School of Medicine Shanghai China; ^2^ School of Public Health Shanghai Jiao Tong University Shanghai China; ^3^ School of Public Health Shanghai Jiao Tong University School of Medicine Shanghai China; ^4^ Department of Clinical Nutrition, Ruijin Hospital Shanghai Jiao Tong University School of Medicine Shanghai China; ^5^ Ruijin Hospital Geriatrics Center Shanghai China

**Keywords:** community‐dwelling, dietary quality, older adults, social support

## Abstract

Diet quality and social support play essential roles in maintaining the health of older adults. However, the relationship between these factors remains uncertain. This study aimed to investigate the association between social support and dietary quality among community‐dwelling older adults in China. This study analyzed 515 community‐dwelling older adults from three districts in Shanghai that were selected based on their geographic location and level of economic development between March and November 2022. Dietary quality was assessed using the China Elderly Dietary Guideline Index (CDGI), China Healthy Eating Index (CHEI), and Dietary Inflammatory Index (DII). Social support was measured using the Social Support Rating Scale (SSRS). Associations between dietary quality and social support were examined using linear regression models. Participants had a mean age of 71.3 ± 4.7 years, and 39.4% were male. The mean CDGI, CHEI, and DII scores were 76.35 ± 11.38, 63.87 ± 9.75, and 0.57 ± 1.65, respectively. The mean SSRS score was 35.05 ± 7.54, ranging from 30.86 to 105.61. Correlation analysis showed that CDGI positively correlated with SSRS and CHEI but negatively correlated with DII. Participants in the highest CDGI tertile reported lower total fat intake and higher consumption of anti‐inflammatory foods and nutrients compared to those in the lowest tertile. After adjusting for confounding factors, a higher CDGI score was significantly associated with higher SSRS scores (*β* = 0.094, 95% CI: 0.056–1.679, *p* = 0.036). Conversely, a lower DII score was significantly associated with higher SSRS scores (*β* = −0.088, 95% CI: −1.587 to −0.030, *p* = 0.042). These results showed that dietary quality and social support are positively correlated among older Chinese adults, highlighting the importance of strengthening social networks to promote healthier diets. These findings underscore the potential for community‐based interventions targeting social and nutritional factors to be correlated with improved health outcomes in aging populations.

## Graphical abstract

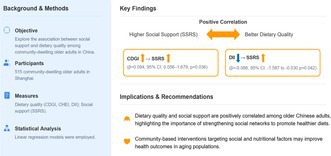

## 1 Background

The aging population is a significant global trend, and by 2050, the number of people aged 65 and over is projected to exceed 1.5 billion, accounting for 16% of the world's population (Chang et al. 2019). By that time, China's population aged 65 years and older is expected to reach 400 million, with 150 million of them aged 80 years and over (Fang et al. 2015). This rapid demographic change will strain healthcare and public health systems significantly. Optimizing diet quality and strengthening social support may be key interventions to cut chronic disease risks and improve the elderly's well‐being.

A nutritious and balanced diet is essential for preserving general well‐being and provides the foundation for the body to sustain normal physiological activities. Certain dietary patterns, such as the Mediterranean and plant‐based diets, have been linked to a reduced risk of cardiovascular diseases (Ellingsen et al. 2008), cognitive decline (van de Rest et al. 2015; Milte and McNaughton 2016), type 2 diabetes mellitus (Jannasch et al. 2017; Qian et al. 2019), and depression (Chan et al. 2019; Matison et al. 2021), as well as enhanced quality of life (Govindaraju et al. 2018). To encourage healthy dietary behaviors, several countries have developed dietary guidelines and dietary indicators based on scientific evidence and local eating habits (Yang et al. 2018). These indicators have also been utilized to explore the relationship between dietary quality and health outcomes. Among these, the China Elderly Dietary Guideline Index (CDGI) was specifically designed based on the China Dietary Guidelines and the Dietary Reference Intakes for Chinese People (Wang et al. 2019). A higher CDGI score reflects better dietary quality (Zhang, Wang, et al. 2021), underscoring its importance as a tool for assessing and promoting healthier eating patterns among the older people in China.

Social support, defined as material and emotional assistance from family, friends, and social networks, plays a vital role in promoting health and well‐being (Li et al. 2022). While the positive causal relationship between social support and health is well established, its influence on crucial health‐related behaviors, such as dietary quality, remains underexplored, particularly among the aging population. In recent years, there has been growing interest in understanding the role of social support in shaping dietary behavior (Teleki et al. 2019; Siopis et al. 2021; Yoshikawa et al. 2021). Social isolation has been shown to increase the risk of malnutrition (Boulos et al. 2017; Gilham et al. 2020), whereas social support may facilitate healthier eating habits among older adults (Bloom et al. 2017). However, the relationship between social support and overall dietary quality remains inconclusive. A study of middle‐aged and older adults in Europe reached a relevant conclusion. Social isolation—defined as living alone or having limited contact with friends and family—was not consistently linked to poor dietary habits, such as low fruit and vegetable intake, across different populations (Delerue Matos et al. 2021). Similarly, a systematic review drew a relevant conclusion. In some cases, social support exerted no clear effect on dietary behavior changes, whereas individual motivation, knowledge, and environmental influences played more prominent roles in adherence to dietary recommendations (Deslippe et al. 2023).

In China, where aging populations often rely on family and community networks for support, understanding the link between social support and dietary quality is particularly important. This study aims to investigate the association between dietary quality and social support among community‐dwelling older adults in China. By utilizing the CDGI as a primary measure of dietary quality, this research seeks to address existing gaps and provide insights into the psychosocial determinants of dietary behavior, with implications for future interventions targeting older adults.

## 2 Methods

### 2.1 Study Design and Population

This cross‐sectional investigation was carried out between March and November 2022. Three districts in Shanghai were chosen, taking into account their geographic distribution and economic development status, from the city's 16 districts. Within each selected district, one town was randomly picked, and subsequently, two to three communities were chosen from each town. The criteria for participant inclusion were: (1) age 65 years or older; (2) ability to independently complete the necessary tests and evaluations; and (3) provision of signed informed consent. Exclusion criteria included: (1) significant speech difficulties and (2) severe restrictions in daily living activities. Initially, 799 older adults were enrolled. However, 236 participants who did not finish the dietary assessment, 7 who could not undergo body composition analysis, and 41 with incomplete anthropometric data were excluded, amounting to 286 individuals. Consequently, 515 older adults living in the community were included in the final analysis. The necessary sample size was determined based on the anticipated relationship between dietary quality and social support. Utilizing the Social Support Rating Scale (SSRS), the average score and standard deviation were projected to be 35.05 ± 7.54, based on previous research. The sample size calculation was based on a minimum detectable difference of 2.0 points on the SSRS. A significance level (*α*) of 0.05 (two‐tailed) and a statistical power (1−*β*) of 0.80 were applied. The analysis indicated that a minimum of 112 participants was required. After considering a potential 20% dropout rate, the adjusted sample size was raised to 140 participants. The study ultimately included 515 participants. This sample size significantly exceeded the minimum threshold, thereby ensuring sufficient statistical power.

The study protocol received ethical approval from the Human Ethics Research Committee of the School of Public Health at Shanghai Jiao Tong University School of Medicine (Approval No. SJUPN202008). Prior to the commencement of the study, written informed consent was secured from all enrolled participants.

### 2.2 Dietary Assessment

A standardized semi‐quantitative Food Frequency Questionnaire (FFQ), consisting of 70 food‐related items, was employed to evaluate participants' dietary habits via in‐person interviews (Bian, Xuan, et al. 2023). The food items were grouped into 14 categories according to their nutritional characteristics. For each item, the FFQ captured three aspects: whether the food was consumed, the typical consumption frequency (times per day, week, month, or year), and the estimated portion size using local units such as ‘liang’ for weight (1 liang = 50 g) or standard cups for volume (1 cup = 250 mL). The research team conducting the dietary assessments comprised experienced dietitians with over 5 years of practice in public hospitals, all of whom underwent specific training before the study. To enhance the precision of participants' food recall, visual aids such as food models and images were utilized. The average daily intake of oil, salt, and sugar was estimated based on the total household consumption of these condiments. All food data were converted to daily intake per individual, and nutrient intakes, including energy, protein, and carbohydrates, were computed using the Chinese Food Composition Table (National Institute of Nutrition and Food Safety, China CDC).

### 2.3 China Elderly Dietary Guideline Index, CDGI

The China Elderly Dietary Guideline Index was developed based on the China Dietary Guidelines and the Dietary Reference Intakes for Chinese People (Wang et al. 2019). The components of the Dietary Guideline Index were categorized into three main groups: (1) the “adequate intake” group, which includes cereals, potatoes, fruits, and vegetables; (2) the “moderate intake” group, comprising aquatic products, livestock, poultry, and eggs; and (3) the “limited intake” group, covering oil, salt, and alcohol. A total of 13 food evaluation indicators were ultimately selected. The CDGI (Chinese Dietary Guideline Index) total score ranges from 0 to 110, derived by aggregating the scores of each individual component. A higher total score indicates a better overall diet quality (Zhang, Wang, et al. 2021).

### 2.4 Chinese Health Eating Index, CHEI

The China Healthy Eating Index (CHEI) was developed in 2017 (Yuan et al. 2017). Its development approach drew on the principles outlined in the Chinese Dietary Guidelines (CDG‐2016). These guidelines served as the index's primary reference (Yang et al. 2018). The Chinese Dietary Guidelines (CDG‐2016) advocate for a balanced dietary pattern as the optimal nutritional model for the Chinese population. Specifically, adults in China are encouraged to consume a minimum of 12 distinct food items daily. Emphasis is placed on increasing the intake of whole grains, vegetables, fruits, fish, and seafood, while limiting the consumption of salt, cooking oil, refined grains, and red meat. The Chinese Healthy Eating Index (CHEI) assesses an individual's compliance with this balanced dietary pattern by evaluating 17 components, comprising 12 adequacy‐related and 5 moderation‐related items. Several of these components align with international dietary indices. For instance, the CHEI shares seven common elements with the AHEI‐2010, including alcohol, red meat, sodium, nuts and legumes, fruits, vegetables, and whole grains. The CHEI is scored on a scale of 0 to 100, where a score of 100 reflects complete adherence to the guidelines, and a score of 0 indicates no adherence whatsoever. A higher CHEI score signifies better alignment with the latest Dietary Guidelines for Chinese People, and its validity and reliability have been thoroughly validated (Wu et al. 2022). It is generally accepted that the CHEI is a suitable tool for measuring dietary quality in general and special Chinese populations.

### 2.5 Dietary Inflammatory Index, DII

The DII was created by Shivappa et al. (Shivappa et al. 2014; Phillips et al. 2019) following an extensive review of research literature involving participants from 11 countries spanning four continents. This research examined the impact of 45 distinct food parameters on six inflammatory markers, including IL‐1*β*, IL‐4, IL‐6, IL‐10, TNF‐*α*, and CRP. In the current study, 28 of these 45 food parameters were utilized to compute the DII. These parameters encompassed carbohydrates, cholesterol, energy, fiber, folate, total fat, iron, magnesium, niacin, monounsaturated fatty acids, n‐3 fatty acids, n‐6 fatty acids, protein, polyunsaturated fatty acids, riboflavin, saturated fats, selenium, thiamine, vitamin A, vitamin C, vitamin D, vitamin E, vitamin B12, vitamin B6, zinc, isoflavones, alcohol, and *β*‐carotene. A comprehensive description of the DII calculation methodology has been provided in prior publications (Bian et al. 2022).

### 2.6 Assessment of Social Support

The level of social support was assessed using the Social Support Rating Scale (SSRS), a tool designed to measure the extent of support individuals receive from family, friends, and their broader social environment (Zhan et al. 2022; Bian, Li, et al. 2023). The SSRS comprises three distinct dimensions: subjective support (evaluating the availability of assistance, relationships with neighbors and colleagues, and the degree of family support), objective support (assessing sources of practical help during emergencies and emotional comfort in stressful or challenging situations), and support utilization (examining how individuals communicate their needs, seek assistance, and engage in group activities when facing difficulties). The total SSRS score is calculated by summing the scores from these three subscales, with a possible range of 12–66. A higher total score reflects a greater level of perceived social support (Xiao et al. 2017).

### 2.7 Covariates

Socio‐demographic characteristics collected in the study comprised age, gender, and educational attainment, which were classified into four groups: high school education or lower, college diploma, bachelor's degree or higher, and master's degree or higher. Health‐related variables included body mass index (BMI), handgrip strength, scores from the Short Physical Performance Battery (SPPB), Morse Fall Scale scores, presence of cognitive impairment, depression, hypertension, diabetes, coronary heart disease, and cancer.

### 2.8 Statistical Analysis

Continuous variables were presented as means ± standard deviations (SD), while categorical variables were reported as frequencies and percentages. For continuous data, comparisons were made using analysis of variance (ANOVA) or independent *t* tests, whereas chi‐square tests were applied for categorical data. Spearman's correlation coefficient was utilized to assess associations between variables. The association between social support and dietary quality was examined using linear regression models. Multivariate regression analyses adjusted for potential confounders, such as age, sex, educational attainment, living arrangement (living alone), household income, body mass index (BMI), cognitive impairment, depression, diabetes, hypertension, coronary heart disease, and cancer. Multicollinearity was evaluated using variance inflation factors (VIF). Sensitivity analyses were performed to ensure the robustness of the results by excluding individuals with missing data. A two‐tailed *p* < 0.05 was deemed statistically significant. All statistical procedures were conducted using SPSS version 22.0.

## 3 Results

### 3.1 Characteristics of the Participants

The general characteristics of the study participants are shown in Table 1. A total of 203 males and 312 females participated, with a mean age of 71.31 ± 4.71 years. The participants' mean ± SD and range of CDGI scores were 76.35 ± 11.38 and 32.10–87.79, respectively. The mean ± SD and range of CHEI scores were 63.87 ± 9.75 and 32.10–87.79, respectively. As CDGI scores increased, CHEI scores tended to rise, while DII scores tended to decrease. The mean ± SD and range of SSRS scores were 35.05 ± 7.54 and 30.86–105.61, respectively. The mean SSRS scores for participants in the first, second, and third tertiles of the CDGI were 33.41 ± 8.01, 35.53 ± 7.68, and 36.23 ± 6.62, respectively. Participants in the highest tertile of the CDGI score showed a lower percentage of males, fewer with a high school education or lower, lower levels of social support and depression, as well as lower height, weight, FFMI, ASMI, and DII scores, but higher SSRS and CHEI scores (*p* < 0.05).

**TABLE 1:** Characteristics of individuals according tertiles of CDGI.

Variable	Tertile 1 (*n* = 172)	Tertile 2 (*n* = 172)	Tertile 3 (*n* = 171)	*p*
Age	70.93 ± 4.93	71.11 ± 4.44	71.90 ± 4.65	0.126
Gender				0.003
Male	79 (45.9)	74 (43.0)	50 (29.2)	
Female	93 (54.1)	98 (57.0)	121 (70.8)	
Education level				< 0.001
High school and below	16 (9.3)	11 (6.4)	7 (4.1)	
College degree	129 (75.0)	97 (56.4)	110 (64.3)	
Bachelor's degree	19 (11.0)	41 (23.8)	32 (18.7)	
Master's degree and above	8 (4.7)	23 (13.4)	22 (12.9)	
Annual household income (RMB)				0.159
≤ 30,000	17 (9.9)	12 (7.0)	15 (8.8)	
30,000–90,000	134 (77.9)	127 (73.8)	133 (77.8)	
90,000–240,000	21 (12.2)	28 (16.3)	22 (12.9)	
> 240,000	0 (0)	5 (2.9)	1 (0.6)	
Lives alone				0.275
Yes	18 (10.5)	10 (5.8)	13 (7.6)	
No	154 (89.5)	162 (94.2)	158 (92.4)	
BMI (kg/m^2^)	23.92 ± 3.45	23.70 ± 3.08	23.12 ± 3.03	0.058
ASMI (kg/m^2^)	6.89 ± 0.99	7.00 ± 1.04	6.67 ± 1.02	0.012
Handgrip strength (kg)	25.90 ± 7.68	26.80 ± 8.62	25.34 ± 7.78	0.240
SSRS score	33.41 ± 8.01	35.53 ± 7.68	36.23 ± 6.62	0.001
SPPB score	10.92 ± 1.70	11.16 ± 1.45	11.26 ± 1.28	0.104
Cognitive impairment				0.060
Yes	57 (33.1)	69 (40.1)	48 (28.1)	
No	115 (66.9)	103 (59.9)	123 (71.9)	
Depression				0.001
Yes	24 (14.0)	11 (6.4)	6 (3.5)	
No	148 (86)	161 (93.6)	165 (96.5)	
T2DM				0.675
Yes	31 (18.0)	27 (15.7)	33 (19.3)	
No	141 (82.0)	145 (84.3)	138 (80.7)	
Hypertension				0.622
Yes	82 (47.7)	73 (42.2)	77 (45.0)	
No	90 (52.3)	99 (57.6)	94 (55.0)	
CVD				0.812
Yes	20 (11.6)	24 (14.0)	22 (12.9)	
No	152 (88.4)	148 (86.0)	149 (87.1)	
Cancer				0.075
Yes	7 (4.1)	1 (0.6)	3 (1.8)	
No	165 (95.9)	171 (99.4)	168 (98.2)	
Low SPPB				0.222
Yes	25 (14.5)	18 (10.5)	15 (8.8)	
No	147 (85.5)	154 (89.5)	156 (91.2)	
Sarcoponia				0.581
Yes	25 (14.5)	19 (11.0)	20 (11.7)	
No	147 (85.5)	153 (89.0)	151 (88.3)	
CDGI score	63.56 V8.12	77.73 ± 2.56	87.82 ± 4.31	< 0.001
DII score	1.18 ± 1.68	0.40 ± 1.58	0.12 ± 1.51	< 0.001
CHEI score	56.46 ± 9.23	65.41 ± 7.68	69.78 ± 7.03	< 0.001

*Note:* Continuous variables are expressed as mean ± standard deviation (SD), while categorical variables are summarized using frequencies (*n*). For statistical analysis, one‐way analysis of variance (ANOVA) was applied to continuous variables, and the Chi‐square test was utilized for categorical variables. A low SPPB score is defined as ≤ 9.

Abbreviations: ASMI, Appendicular Skeletal Muscle Index; BMI, body mass index; CDGI, Chinese Dietary Guidelines index for the elderly; CHEI, Chinese healthy eating index; CVD, cardiovascular disease; DII, dietary inflammatory index; FFMI, fat‐free mass index; FMI, fat mass index; SPPB, Short Physical Performance Battery; SSRS, Social Support Rating Scale; T2DM, type 2 diabetes mellitus.

### 3.2 Nutrient and Food Intake According to CDGI Tertiles

Nutrient and food intake according to tertiles of CDGI are shown in Table 2. The mean total energy intake for participants in the first, second, and third tertiles of the CDGI was 1413.29 ± 466.05, 1371.71 ± 375.24, and 1336.63 ± 367.9 kcal, respectively (*p* = 0.216), while carbohydrate intake was 154.95 ± 56.92, 164.75 ± 51.21, and 165.91 ± 54.32 g (*p* = 0.121), protein intake was 52.76 ± 20.21, 57.71 ± 19.47, and 56.94 ± 16.65 g (*p* = 0.033), and total fat intake was 66.36 ± 27.74, 58.99 ± 21.02, and 54.41 ± 19.70 g (*p* < 0.001). Compared to participants in the first tertile, those in the highest tertile of the CDGI showed lower intakes of total fat and higher intakes of anti‐inflammatory foods and nutrients, such as protein, fiber, PUFA, MUFA, n‐3 and n‐6 fatty acids, *β*‐carotene, vitamins, folic acid, magnesium, and zinc (*p* < 0.05).

**TABLE 2:** Nutrient and food intake according to tertiles of CDGI.

Variable	All	Tertile 1 (*n* = 172)	Tertile 2 (*n* = 172)	Tertile 3 (*n* = 171)	*p*
Total energy (kcal)	1373.95 ± 406.02	1413.29 ± 466.05	1371.71 ± 375.24	1336.63 ± 367.9	0.216
Carbohydrate (g)	161.86 ± 54.32	154.95 ± 56.92	164.75 ± 51.21	165.91 ± 54.32	0.121
Protein (g)	55.80 ± 18.93	52.76 ± 20.21	57.71 ± 19.47	56.94 ± 16.65	0.033
Total fat (g)	59.93 ± 23.57	66.36 ± 27.74	58.99 ± 21.02	54.41 ± 19.7	< 0.001
Saturated fat (g)	9.64 ± 5.97	9.4 ± 6.52	9.80 ± 6.63	9.73 ± 4.57	0.807
Fiber (g)	10.29 ± 4.12	8.95 ± 4.21	10.87 ± 4.39	11.06 ± 3.38	< 0.001
PUFA (g)	4.21 ± 2.51	3.48 ± 2.39	4.32 ± 2.39	4.81 ± 2.57	< 0.001
MUFA (g)	6.90 ± 2.93	6.1 ± 2.75	7.1 ± 3.22	7.49 ± 2.61	< 0.001
n‐3 Fatty acids (g)	0.39 ± 0.25	0.34 ± 0.22	0.4 ± 0.26	0.43 ± 0.26	0.005
n‐6 Fatty acids (g)	4.76 ± 3.26	4.09 ± 3.57	5.05 ± 3.41	5.16 ± 2.62	0.003
Vitamin B1 (mg)	0.70 ± 0.33	0.64 ± 0.35	0.73 ± 0.34	0.71 ± 0.31	0.037
Vitamin B2 (mg)	0.85 ± 0.34	0.76 ± 0.31	0.89 ± 0.42	0.9 ± 0.24	< 0.001
Vitamin B6 (mg)	24.83 ± 60.39	23.62 ± 41.61	28.15 ± 89.18	22.71 ± 35.59	0.671
Vitamin B12 (μg)	3.29 ± 9.56	3.99 ± 14.42	2.76 ± 4.54	3.11 ± 6.76	0.466
Vitamin C (mg)	86.35 ± 42.68	77.70 ± 40.16	90.4 ± 47.04	90.97 ± 39.3	0.005
*β*‐Carotene (μg)	8544.11 ± 4893.41	7568.11 ± 4552.98	9014.46 ± 5150.37	9052.71 ± 4839.22	0.006
Vitamin D (μg)	4.02 ± 1.96	3.48 ± 1.91	4.1 ± 1.99	4.49 ± 1.85	< 0.001
Vitamin E (mg)	10.81 ± 4.50	9.51 ± 4.53	11.32 ± 4.48	11.61 ± 4.2	< 0.001
Niacin (mg)	12.74 ± 4.86	12.63 ± 5.48	13 ± 4.6	12.6 ± 4.45	0.697
Folic acid (μg)	294.24 ± 118.05	263.97 ± 111.87	310.31 ± 128.72	308.52 ± 107.17	< 0.001
Isoflavones (mg)	6.53 ± 7.21	5.78 ± 7.29	6.94 ± 7.06	6.87 ± 7.28	0.247
Mg (mg)	281.49 ± 95.32	252.56 ± 91.7	292.28 ± 99.15	299.73 ± 88.46	< 0.001
Se (mg)	32.25 ± 13.29	31.13 ± 15.48	32.89 ± 13.16	32.72 ± 10.82	0.401
Zn (mg)	10.07 ± 3.46	9.21 ± 3.43	10.47 ± 3.92	10.52 ± 2.78	< 0.001

Abbreviations: MUFA, monounsaturated fatty acid; PUFA, polyunsaturated fatty acid.

### 3.3 Correlation Analysis Between SSRS, CDGI, CHEI, and DII

**FIGURE 1:**
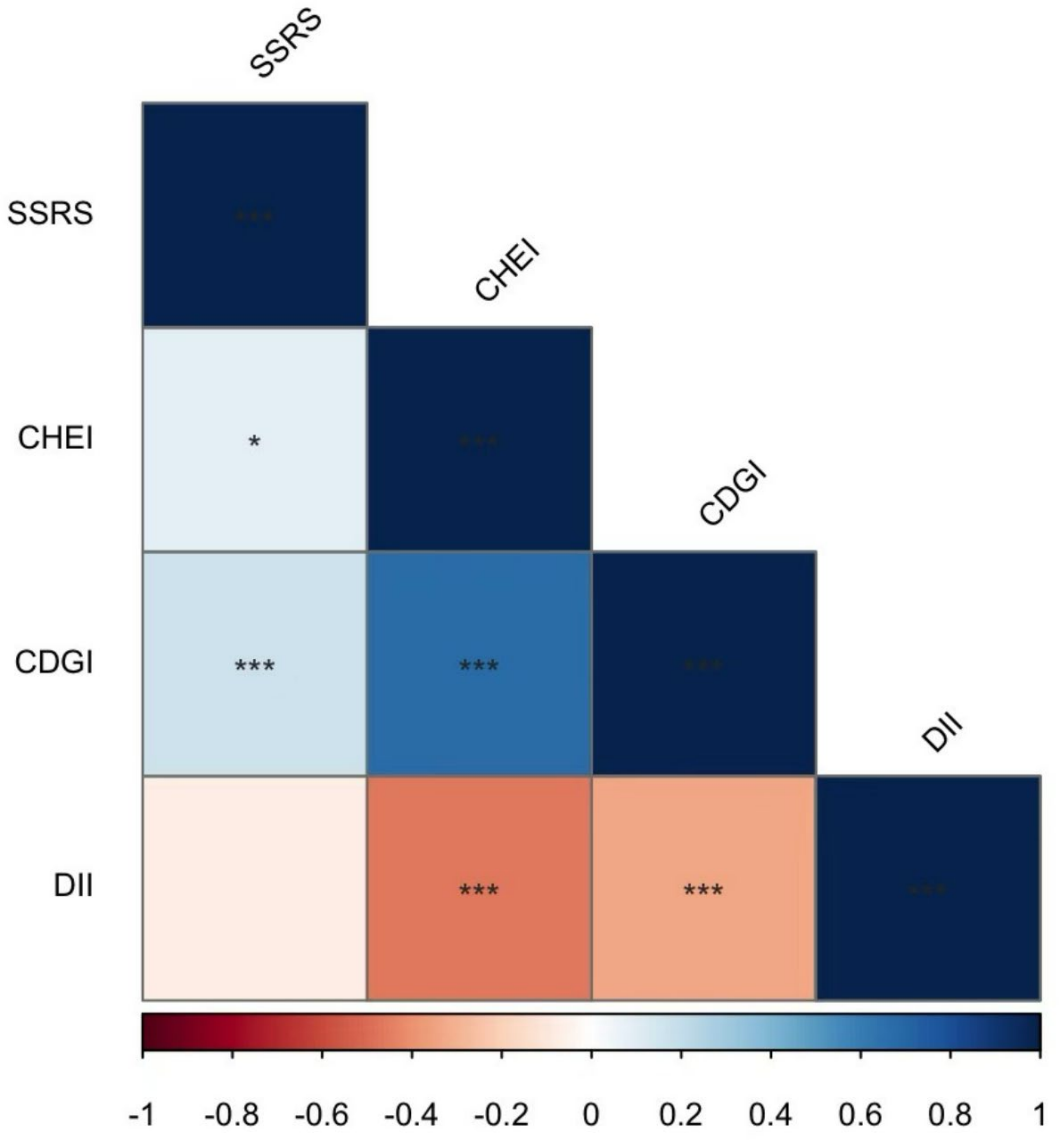
Heat map of the relationships between SSRS, DII, CDGI and CHEI.*p < 0.05, **p < 0.01, ***p < 0.01.

The correlation analysis between SSRS, CDGI, CHEI, and DII suggested that CDGI was positively correlated with SSRS (*r*: 0.169, *p* < 0.001) and CHEI (*r*: 0.676, *p* < 0.001), whereas it negatively correlated with DII (*r*: −0.331, *p* < 0.001). A positive correlation was also observed between the SSRS and CHEI (*r*: −0.331, *p* < 0.05). The results are shown in the heat map in Figure 1.

### 3.4 Association between Dietary Quality and Social Support

The relationship between different dietary quality assessment parameters and social support is shown in Table 3. Both the unadjusted and adjusted models revealed that a higher CDGI (indicating a healthier diet) was associated with a higher level of SSRS (*β* coefficient: 0.117, 95% CI: 0.061, 0.174, *p* < 0.001 in model 1; *β* coefficient: 0.094, 95% CI: 0.056, 1.679, *p* = 0.036 in model 3). In the adjusted model, a higher DII (indicating a more pro‐inflammatory diet) was associated with a lower level of SSRS (*β* coefficient: −0.088, 95% CI: −1.587, −0.030, *p* = 0.042 in model 3). In the unadjusted model, a higher CHEI (indicating healthier eating) was associated with a higher level of SSRS (*β* coefficient: 0.094, 95% CI: 0.006, 0.139, *p* = 0.033 in model 1). However, no significant association was found between CHEI and SSRS in the adjusted model.

**TABLE 3:** Association of dietary quality with SSRS.

Variables	Model 1		Model 2		Model 3	
	*β* (95% CI)	*p*	*β* (95% CI)	*p*	*β* (95% CI)	*p*
DII	−0.070 (−0.718, 0.074)	0.111	−0.053 (−0.642, 0.156)	0.231	−0.088 (−1.587, −0.030)	0.042
CDGI	0.117 (0.061, 0.174)	< 0.001	0.137 (0.032, 0.151)	0.003	0.094 (0.056, 1.679)	0.036
CHEI	0.094 (0.006, 0.139)	0.033	0.067 (−0.016, 0.119)	0.135	0.049 (−0.337, 1.247)	0.259

*Note:* *β* values and 95% confidence intervals were estimated using linear regression models with different levels of adjustment. Model 1: unadjusted. Model 2: adjusted for age, gender, BMI, and education level. Model 3: additionally adjusted for living status (living alone), average household income, cognitive impairment, depression, diabetes, hypertension, coronary heart disease, and cancer.

Abbreviations: CDGI, China Elderly Dietary Guideline Index; CHEI, Chinese Health Eating Index; DII, dietary inflammatory index.

## 4 Discussion

Diet quality and social support play an increasingly important role in the quality of life of older adults. To explore the relationship between these factors, we conducted a cross‐sectional survey using a rigorous sampling design and standardized methods to ensure the reliability of our findings. In this study, dietary quality was assessed using the CDGI, CHEI, and DII, which reflect overall dietary quality and have been well validated in Chinese populations (Wang, Sarker, et al. 2024; Yang et al. 2018; Zhang, Wang, et al. 2021; Wu et al. 2022; Wang, Sun, et al. 2024). Our correlation analyses revealed that CDGI was positively correlated with CHEI and negatively correlated with DII, with significant correlations among all three measures. The mean SSRS score for community‐dwelling older adults aged 65 and older in this study was 35.10 ± 7.54, which is consistent with the results of other studies involving older populations in urban areas of China (Bian, Li, et al. 2023; Shen et al. 2022). After adjusting for confounding factors, our study found that higher CDGI and lower DII scores were associated with higher social support levels, indicating that social support was positively linked to overall dietary quality and negatively correlated with pro‐inflammatory diets. This suggests that higher levels of social support promote better dietary quality. Further analysis revealed that participants in the highest CDGI tertile consumed less total fat, more protein, and a higher intake of anti‐inflammatory foods and nutrients—such as fiber, unsaturated fatty acids, and vitamins while maintaining a similar energy intake (Tables 2 and S1). These findings emphasize that higher CDGI scores were linked to healthier, anti‐inflammatory diets, supporting the idea that high levels of social support are associated with improved dietary quality.

Importantly, low social support and poor dietary quality are linked to chronic inflammation. Previous studies have demonstrated that high social isolation correlates with elevated chronic inflammation markers. These markers include C‐reactive protein (CRP), interleukin‐6 (IL‐6), and soluble urokinase plasminogen activator receptor (suPAR). All of these markers are associated with poorer health outcomes and increased mortality risk (Matthews et al. 2024). Moreover, highly socially isolated individuals show stronger pro‐inflammatory activity when exposed to stress or inflammation. Pro‐inflammatory cytokines can interfere with pathways like the vagus nerve or blood–brain barrier. This interference raises sensitivity to social threats and impairs social connections (Eisenberger et al. 2017). Furthermore, low social support can result in neglect of dietary quality and malnutrition, potentially leading to protein‐energy malnutrition and micronutrient deficiencies, triggering an inflammatory response and perpetuating a vicious cycle (Massironi et al. 2023; Stumpf et al. 2023). Therefore, both low social support and diminished dietary quality can contribute to a systemic inflammatory response, placing older adults in a state of chronic inflammation that causes significant health challenges.

Similar findings were observed in a study conducted in Yunnan, China, which examined the relationship between the Multidimensional Scale of Perceived Social Support (MSPSS) and the Chinese Dietary Balance Index‐16 (DBI‐16) among ethnic minorities in southwest China. The results demonstrated a positive correlation between perceived social support from family, friends, and significant others and dietary quality (Zhang, Ruan, et al. 2021). Similarly, a cross‐sectional study of more than 3000 U.S. adults aged 40 years and older found that social support among middle‐aged and older men was positively associated with the Healthy Eating Index‐2010 (HEI‐2010) (Pieroth et al. 2017). However, this association was not statistically significant among women and minority youth in the United States (Pieroth et al. 2017; Anderson Steeves et al. 2016). These findings suggest that the relationship between social support and dietary quality may vary across age groups and genders. Additionally, a study of middle‐aged and older adults found that loneliness and limited social contact were associated with poorer dietary quality. Specifically, older adults who were lonely consumed fewer vegetables and fruits, were more likely to skip breakfast, and consumed more calories from eating out, whereas those with higher levels of social support demonstrated healthier dietary behaviors (Yoshikawa et al. 2021). Notably, earlier research has reported a decline in social support for older adults in China (Zhao et al. 2020). This decline underscores an urgent need to address the social support systems for Chinese seniors, as inadequate social support is likely to contribute to poor dietary quality, leading to significant negative effects on their overall well‐being.

Previous research has found that the correlation between dietary quality and social support may be primarily due to the facilitating effect of social support on healthy eating behaviors. For instance, a study of middle‐aged and older adults demonstrated that structured social support, such as group discussions and activities, positively influenced participants' dietary habits (Yoshikawa et al. 2021). Similarly, another study revealed that social support could enhance the confidence of patients with type 2 diabetes mellitus in managing their dietary behaviors, thereby promoting healthier food choices (Yang et al. 2021). Psychosocial factors, such as depression, can act as mediating variables, playing a significant role in the relationship between social support and diet quality. Depression is strongly associated with poor dietary quality, as individuals with depression are more likely to choose foods high in fat and sugar and less likely to adhere to healthy dietary patterns, such as the Mediterranean or DASH diets. This may be attributed to depression‐related symptoms, including fatigue, loss of interest, and reduced self‐care. Consequently, an increase in depressive symptoms can directly lower the quality of an individual's diet (Xu et al. 2021). Furthermore, individuals with poor dietary quality are at higher risk of developing major depression compared to those with healthier diets (Wolters et al. 2021). In this study, a lower percentage of depressive symptoms was observed among individuals in the highest tertile of the CDGI, consistent with this relationship. Additionally, high levels of social support significantly reduce depression rates (Gariepy et al. 2016). Strong social support also alleviates depressive symptoms, which, in turn, encourages healthier eating behaviors and is correlated with higher dietary quality (Yoshikawa et al. 2021). These findings suggest that dietary quality, mental health, and social support form a mutually reinforcing positive feedback loop. To better understand the connections among these factors, further longitudinal studies across diverse populations are needed.

A meta‐analysis of 136 studies using the Social Support Rating Scale (SSRS) revealed a decline in social support scores among older adults in China, with decreases of 5.09 and 0.73 standard deviations between 1994 and 2018, respectively (Zhao et al. 2020). This decline may be attributed to the prevalence of the “four‐two‐one” family structure in Chinese families and the preference for “aging‐in‐place” among older individuals, both of which have contributed to reduced levels of social support for older people in recent years. Aligned with these concerns, Healthy People 2030 emphasizes the importance of helping individuals access the social support needed to thrive in all aspects of life, including living, working, learning, and recreation (Rine 2023). To achieve this goal in the context of China, it is essential to optimize aging models for older people. A combined approach that integrates community‐based and home‐based care could be instrumental. This model would increase community engagement opportunities for older adults. It would also promote the community‐based home care model, alleviate family eldercare burdens, and reduce long‐term care costs (He et al. 2023). Moreover, public health strategies with robust health economic outcomes should be strengthened. These include establishing more universities for older adults, highlighting the value of regular parent–child contact, and popularizing dietary guidelines via community programs. Additional initiatives to build social connections and reduce social isolation should also be implemented. Together, these strategies can greatly strengthen the social support network for older adults and boost their overall well‐being.

The limitations of this study should be acknowledged. First, although three communities were included, the sample size was relatively small. As all participants were recruited exclusively from Shanghai, the findings may not be representative of the broader population of older adults in the Shanghai area, nor can they be generalized to older adults in other provinces of China. Furthermore, caution should be exercised when applying these conclusions to international comparisons of social support. Second, the statistical significance of the correlation analyses was limited due to the small sample size. Third, this study employeFd a cross‐sectional design, which prevents the establishment of causal relationships between social support and dietary quality. Future prospective cohort studies are necessary to validate these findings. Additionally, this study did not assess certain biomarkers related to dietary quality, such as inflammatory markers like C‐reactive protein (CRP). Biomarker‐based evaluations could provide more robust evidence for the observed associations. Finally, other potential confounding factors, such as sleep quality and chronic pain, were not considered in the analysis, which may have influenced the results. Further research addressing these variables is needed to strengthen the conclusions of this study.

## 5 Conclusions

Our study found that dietary quality is associated with social support among community‐dwelling older adults in China. Social support showed a positive correlation with the CDGI and a negative correlation with the DII. These findings confirm that social support may play a critical role in enhancing dietary quality and overall health among Chinese older adults.

## Author Contributions

Writing (original draft), Xiaoyan Zhang, Yuanyuan Yan, and Dongsheng Bian; Writing (review and editing), Xiaoyan Zhang, Yuanyuan Yan, Fang Wu, and Dongsheng Bian; Conceptualization, Feika Li, Jialin Liu, Fang Wu, and Dongsheng Bian; Formal analysis, Xiaoyan Zhang and Dongsheng Bian; Funding acquisition, Dongsheng Bian; Methodology, Xiaoyan Zhang and Feika Li; Project administration, Jialin Liu and Dongsheng Bian; Resources, Fang Wu and Dongsheng Bian; Supervision, Feika Li, Jialin Liu, and Fang Wu.

## Funding

This research was supported by the National Natural Science Foundation of China (82304114); Shanghai Municipal Commission of Health (GWVI‐11.2‐YQ36, GWVI‐11.1‐48).

## Ethics Statement

All procedures involving human participants complied with the ethical standards of the Declaration of Helsinki. This study was approved by the Human Ethics Research Committee of the School of Public Health, Shanghai Jiao Tong University School of Medicine (Approval No. SJUPN202008). Written informed consent was obtained from all participants before the study began.

## Consent

The authors have nothing to report.

## Conflicts of Interest

The authors declare no conflicts of interest.

## Supporting information


**Table S1:** Nutrient and food intake according to tertiles of CHEI.

## Data Availability

All data generated or analyzed during this study are included in this published article. Any additional data are available from the corresponding author on reasonable request.
