# Supplementary material for: Social Support as a Determinant of Dietary Quality in Community‐Dwelling Older Adults in China
Source: Food Sci Nutr. 2026 Jan 22;14(1):e71476. doi: 10.1002/fsn3.71476 (PMC12827061; doi:10.1002/fsn3.71476)
Supplement: Supplementary file 1 — Table S1: Nutrient and food intake according to tertiles of CHEI. [file FSN3-14-e71476-s001.docx]

Supplementary Table 1 Nutrient and food intake according to tertiles of CHEI

| Variable | all | Tertile 1 (n=172) | Tertile 2 (n=172) | Tertile 3 (n=171) | p-value |
| --- | --- | --- | --- | --- | --- |
| Total energy (kcal) | 1373.95±406.02 | 1303.63±420.51 | 1309.74±332.73 | 1509.26±426.16 | <0.001 |
| Carbohydrate (g) | 161.86±54.32 | 138.65±48.83 | 158.93±44.92 | 188.15±56.96 | <0.001 |
| Protein (g) | 55.80±18.93 | 47.13±14.30 | 54.93±16.03 | 65.40±21.23 | <0.001 |
| Total Fat (g) | 59.93±23.57 | 64.41±25.08 | 55.03±19.38 | 60.36±25 | 0.001 |
| Saturated fat (g) | 9.64±5.97 | 8.66±5.84 | 9.06±4.39 | 11.22±7.10 | <0.001 |
| Fiber (g) | 10.29±4.12 | 8.28±3.20 | 10.40±3.38 | 12.19±4.68 | <0.001 |
| PUFA (g) | 4.21±2.51 | 3.41±2.37 | 4.06±2.04 | 5.15±2.75 | <0.001 |
| MUFA (g) | 6.90±2.93 | 5.88±2.41 | 6.83±2.32 | 7.98±3.53 | <0.001 |
| n-3 Fatty acids (g) | 0.39±0.25 | 0.31±0.19 | 0.37±0.20 | 0.49±0.30 | <0.001 |
| n-6 Fatty acids (g) | 4.76±3.26 | 3.98±3.56 | 4.57±2.37 | 5.75±3.48 | <0.001 |
| Vitamin B1 (mg) | 0.70±0.33 | 0.58±0.27 | 0.67±0.23 | 0.84±0.41 | <0.001 |
| Vitamin B2 (mg) | 0.85±0.34 | 0.71±0.26 | 0.85±0.24 | 0.99±0.42 | <0.001 |
| Vitamin B6 (mg) | 24.83±60.39 | 20.7±36.24 | 18.05±26.59 | 35.81±93.85 | 0.013 |
| Vitamin B12 (µg) | 3.29±9.56 | 3.2±12.42 | 2.95±7.53 | 3.71±7.99 | 0.76 |
| Vitamin C (mg) | 86.35±42.68 | 71.61±35.65 | 87.72±36.48 | 99.79±49.86 | <0.001 |
| β-Carotene (µg) | 8544.11±4893.41 | 7347.24±4553.23 | 8625.15±4384.77 | 9666.46±5429.87 | <0.001 |
| Vitamin D (µg) | 4.02±1.96 | 3.3±1.86 | 3.98±1.66 | 4.79±2.06 | <0.001 |
| Vitamin E (mg) | 10.81±4.50 | 8.86±3.8 | 10.80±3.66 | 12.78±5.04 | <0.001 |
| Niacin (mg) | 12.74±4.86 | 10.91±4.24 | 12.57±4.30 | 14.75±5.22 | <0.001 |
| Folic acid (µg) | 294.24±118.05 | 250.72±96.77 | 298.1±117.37 | 334.12±123.80 | <0.001 |
| Isoflavones (mg) | 6.53±7.21 | 5.20±6.72 | 5.89±6.05 | 8.51±8.31 | <0.001 |
| Mg (mg) | 281.49±95.32 | 235.16±71.45 | 280.27±80.12 | 329.30±106.81 | <0.001 |
| Se (mg) | 32.25±13.29 | 27.40±9.33 | 31.54±13.22 | 37.85±14.66 | <0.001 |
| Zn (mg) | 10.07±3.46 | 8.46±2.69 | 10.05±2.68 | 11.71±4.04 | <0.001 |
